# Healthcare practitioners’ views of social media as an educational resource

**DOI:** 10.1371/journal.pone.0228372

**Published:** 2020-02-06

**Authors:** Adam G. Pizzuti, Karan H. Patel, Erin K. McCreary, Emily Heil, Christopher M. Bland, Eric Chinaeke, Bryan L. Love, P. Brandon Bookstaver

**Affiliations:** 1 Department of Clinical Pharmacy and Outcomes Sciences, University of South Carolina College of Pharmacy, Columbia, South Carolina, United States of America; 2 Kaiser Permanente Georgia, Atlanta, Georgia, United States of America; 3 University of Wisconsin Health, Madison, Wisconsin, United States of America; 4 University of Maryland College of Pharmacy, Baltimore, Maryland, United States of America; 5 University of Georgia College of Pharmacy, Savannah, Georgia, United States of America; Leiden University Medical Center, NETHERLANDS

## Abstract

Social media is increasingly utilized as a resource in healthcare. We sought to identify perceptions of using social media as an educational tool among healthcare practitioners. An electronic survey was distributed to healthcare administrators, nurses, nurse practitioners, pharmacists, physicians, and physician assistants f hospital systems and affiliated health science schools in Georgia, Maryland, South Carolina, and Wisconsin. Survey questions evaluated respondents’ use and views of social media for educational purposes and workplace accessibility using a Likert scale (1 = strongly disagree, 5 = strongly agree). Nurses (75%), pharmacists (11%), and administrators (7%) were the most frequent respondents. Facebook® (27%), Pinterest® (17%), and Instagram® (17%) were the most frequently accessed social media platforms. Nearly 85% agreed or strongly agreed that social media can be an effective tool for educational purposes. Among those who had social media platforms, 43.0% use them for educational purposes. Pinterest® (30%), Facebook® (22%), LinkedIn® (16%), and Twitter® (14%) were most frequently used for education. About 50% of respondents had limited or no access to social media at work. Administrators, those with unlimited and limited work access, and respondents aged 20–29 and 30–39 years were more likely to agree that social media is an educational tool (OR: 3.41 (95% CI 1.31 to 8.84), 4.18 (95% CI 2.30 to 7.60), 1.66 (95% CI 1.22 to 2.25), 4.40 (95% CI 2.80 to 6.92), 2.14 (95% CI 1.53 to 3.01) respectively). Residents, physicians, and those with unlimited access were less likely to agree with allowing social media access at work for educational purposes only. Healthcare practitioners frequently utilize social media, and many believe it can be an effective educational tool in healthcare.

## Introduction

Social media is an effective communication tool allowing people to connect and share information [1]. Approximately 75% of online Americans are influenced by information on social media [2]. Social media platforms have grown into a habitual activity for many, including healthcare professionals. While the perception for negative impact on productivity and efficiency may exist, many use social media as a tool for program marketing, research dissemination, and education and training [3–12]. Social media platforms such as Twitter® have been used in the educational curriculum of medical training programs, increasing access to key resources and content knowledge [13,14]. Many leading healthcare organizations and medical expert groups link conference attendees, members and others using a Twitter hashtag (#) chat to educate and discuss current and controversial topics [15,16, 45]. Given the influx of professional users and benefit to intended audience, several groups have published guidance on how healthcare practitioners and institutions may use social media as a positive platform for marketing and disseminating scholarly deliverables [17,18].

Healthcare information is constantly changing as new clinical evidence becomes available. It is suggested that clinical practice guideline recommendations are often outdated within 6 years of publication [19]. Social media platforms have the potential to aid the individual practitioner in notifications of newly published evidence and pipeline data. Many journals have turned to social media to disseminate updates and healthcare information to end users [20–22]. While this could be a potential outlet for acquiring or alerting to new, evidence-based information, many institutions limit or block access to social media in the workplace [23]. This decision may be because of the lack of awareness of social media’s potential benefits and use by healthcare workers. The purpose of this study was to assess healthcare practitioners’ views on and the use of social media for educational purposes.

## Materials and methods

The study protocol was reviewed by the University of South Carolina Institutional Review Board (IRB) and determined not to meet the criteria for human subjects research; therefore, this study received exempt status. This was a cross-sectional, survey-based study conducted at four health sciences colleges (University of Georgia, University of Maryland, University of South Carolina, and the University of Wisconsin) and affiliated hospitals within the United States. The primary study objective was to measure and compare attitudes regarding social media platforms use for educational purposes among healthcare professionals. The survey instrument consisted of 70-items developed by two authors (AP, KP) with input from all co-authors. Survey items were comprised of various question formats primarily of Likert scale type (1 = strongly agree, 5 = strongly disagree). Participants were asked to provide insight into their quantitative and qualitative use of social media, attitudes regarding social media use for educational purposes and stances regarding social media use in the workplace. The survey also included a series of demographic questions (e.g. position, time since terminal training and geographic location). Educational use of social media was defined at the beginning of the survey as anything regarding the healthcare field that you deem as knowledgeable and useful information (e.g. accessing journal articles, reading drug updates) (S1 File: Survey PDF). Respondents who have a split position (e.g. clinical faculty) were asked to answer the questions related to social media access from the perspective of their institution where patient care activities primarily occur.

The survey was piloted among health science faculty excluded from the final study population. Feedback resulted in condensing the survey to increase likelihood of survey completion. The estimated survey completion time was 5 to 10 minutes (S1 File: Survey PDF). The survey was created and electronically administered using REDCap® (Vanderbilt University–Nashville, TN, 7.5.2, 2017) beginning January 2018 [24]. Licensed healthcare practitioners and administrators were the target population. Residents were intended to be physician or pharmacist trainees currently in a post-graduate residency program. Students were excluded from the survey. Branching logic was used to allow questions to be visible based on previous answers regarding which social media platform they use. Respondents received up to three email reminders until survey closure in May 2018. Survey respondents remained anonymous but were offered the option to enter a random drawing for an incentive upon survey completion. Co-investigators from each of the included sites were responsible for ensuring distribution to targeted healthcare practitioners.

### Statistical analysis

Responses were analyzed to compare how different healthcare professionals are using social media and to identify the potential for educational use. Bivariate analyses were performed to examine unadjusted variation between professional affiliation and each of the various covariates (e.g. age, access) in the survey. We conducted multivariable logistic regression to evaluate the likelihood of professionals to agree or disagree with each of the survey questions: (1) social media is an effective educational tool and (2) social media should be accessed at the workplace for educational purposes only in separate models. We chose the Likert-style question stating social media is an effective tool for educational purposes to fulfill the analysis of what factors contribute to those who use social media for educational purposes. The confounders controlled for in this analysis were age, social media access at work, and profession type. These were Likert-style questions transformed to agree (yes), for all responses that were strongly agree and agree, or disagree (no), for all the responses that were strongly disagree and disagree. The answers for neither disagree or agree were not used.

To account for potential selection bias due to unbalanced nurse professional response, we conducted a propensity-weighted logistic regression using age and geographical location to define the weighting class which stabilized the unbalanced nurse professional’s response. Higher propensity weights were assigned to subjects with low responses and vice versa. It is assumed that one weighting adjustment is enough to address non-response bias in all estimates [25, 26]. A p-value of < 0.05 was deemed statistically significant. All analyses were conducted in SAS version 9.4.

## Results

**Fig 1:**
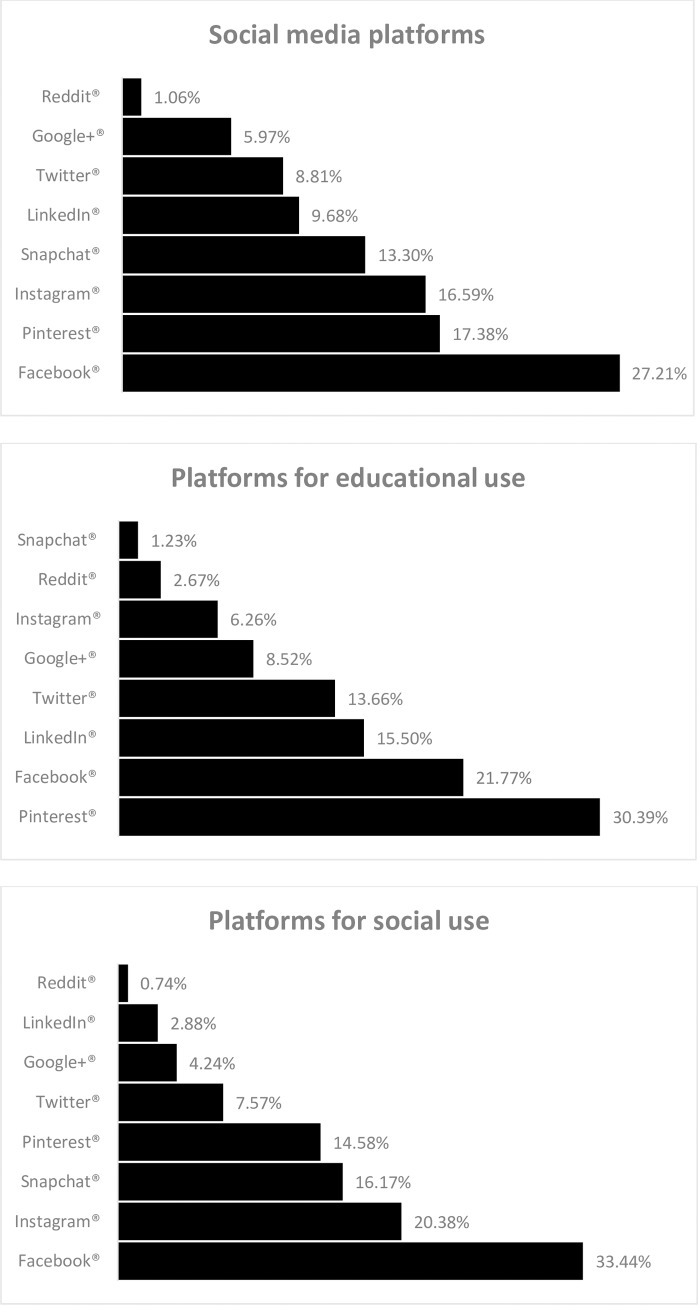
Survey responses to what current social media account they have and their current use of social media by platforms.

There were 1,644 initial responses, with 141 excluded due to respondents’ primary professional role(s) not being an active healthcare practitioner. Most respondents were nurses, followed by pharmacists, administrators and physicians. Among respondents, 53% were under the age of 40 and 60% had worked for 10 years or less in their current role (Table 1). Of respondents with social media accounts, 43% reported using it for educational purposes, but there was a higher percent of those who agreed that social media could be an effective educational tool (85%) (Table 2). Facebook® (27.2%) was the most commonly used social media platform for any purpose, followed by Pinterest® (17.4%) and Instagram® (16.6%). The social media platforms used for educational purposes differed however, as Pinterest®, Facebook®, LinkedIn® and Twitter® were the four most frequently used platforms (Fig 1). Table 2 shows the use of social media for educational purposes by profession and the bivariate analysis results.

**Table 1:** Baseline characteristics of respondents and social media handle.

		Frequency	Percentage
**Age (n = 1,501)**			
	20–29	359	23.92
	30–39	458	30.51
	40–49	316	21.05
	50–59	269	17.92
	60–69	92	6.13
	70–79	5	0.33
	>80	2	0.14
**Education Years (n = 1,466)**			
	≤ 5 Years	557	37.99
	6–10 Years	318	21.69
	11–15 Years	183	12.48
	16–20 Years	133	9.07
	≥ 21 Years	275	18.77
**Professional Affiliation (n = 1,608)**			
	Registered Nurses	1113	74.85
	Pharmacists	162	10.89
	Administrators	98	6.59
	Attending physicians	58	3.90
	Residents	56	3.77
**Geography (n = 1,481)**			
	Wisconsin	828	55.91
	South Carolina	492	33.22
	Others	118	7.97
	Maryland	23	1.55
	Georgia	20	1.35
**Social media access at work place (n = 1,500)**			
	Unknown	595	39.66
	Limited access	505	33.67
	No access	255	17.00
	Unlimited access	145	9.67
**How respondents access social media at work (n = 1,408)**			
	Personal phone/computer	840	59.7
	I do not access it at work	506	35.9
	Work phone/computer	41	2.9
	Other	21	1.5
**How respondents use their social media accounts (n = 2,356)**			
	Social	1347	57.17
	Educational	645	27.38
	Others	364	15.45

**Table 2:** General results by profession.

		Professional affiliation														
		Residents			Admin			Pharmacists			Nurses			Physicians		
		N	%	p	N	%	p	N	%	p	N	%		N	%	p
**Age**				< .0001			0.0014			0.0019			0.1814			< .0001
	20–29	36	66.67		7	7.22		38	23.60		277	25.02		0	0.00	
	30–39	16	29.63		31	31.96		66	40.99		327	29.54		14	24.56	
	40–49	2	3.70		33	34.02		33	20.50		222	20.05		23	40.35	
	50–59	0	0.00		18	18.56		11	6.83		209	18.88		16	28.07	
	60–69	0	0.00		8	8.25		13	8.07		67	6.05		3	5.26	
	70–79	0	0.00		0	0.00		0	0.00		3	0.27		1	1.75	
	>80	0	0.00		0	0.00		0	0.00		2	0.18		0	0.00	
**Education years**				< .0001			0.0144			0.1808			0.4855			< .0001
	≤ 5 Years	50	92.59		26	28.57		49	30.82		407	37.41		6	10.53	
	6–10 Years	2	3.70		17	18.68		43	27.04		240	22.06		13	22.81	
	11–15 Years	1	1.85		21	23.08		24	15.09		135	12.41		8	14.04	
	16–20 Years	1	1.85		11	12.09		12	7.55		93	8.55		16	28.07	
	≥ 21 Years	0	0.00		16	17.58		31	19.50		213	19.58		14	24.56	
**Social media platforms**																
**Facebook**																
	No	11	19.64	0.8452	14	14.29	0.1070	25	15.43	0.0823	137	12.31	< .0001	16	27.59	0.1862
	Yes	45	80.36		84	85.71		137	84.57		976	87.69		42	72.41	
**Twitter**																
	No	30	53.57	0.0003	60	61.22	0.0022	79	48.77	< .0001	852	76.55	0.0029	34	58.62	
	Yes	26	46.43		38	38.78		83	51.23		261	23.45		24	41.38	0.0053
**LinkedIn**																0.0489
	No	38	67.86	0.5073	48	48.98	< .0001	77	47.53	< .0001	828	74.39	0.0006	35	60.34	
	Yes	18	32.14		50	51.02		85	52.47		285	25.61		23	39.66	
**Snapchat**																
	No	22	39.29	0.0006	67	68.37	0.1360	104	64.20	0.4178	612	54.99	< .0001	49	84.48	0.0002
	Yes	34	60.71		31	31.63		58	35.80		501	45.01		9	15.52	
**Google+**																
	No	47	83.93	0.7901	77	78.57	0.2776	137	84.57	0.4872	906	81.40	0.0627	46	79.31	0.5006
	Yes	9	16.07		21	21.43		25	15.43		207	18.60		12	20.69	
**Instagram**																
	No	18	32.14	0.0030	48	48.98	0.5865	80	49.38	0.5444	508	45.64	< .0001	41	70.69	0.0031
	Yes	38	67.86		50	51.02		82	49.38		605	54.36		17	29.31	
**Pinterest**																
	No	30	53.57	0.5184	56	57.14	0.1107	72	44.44	0.1901	449	40.34	< .0001	49	84.48	< .0001
	Yes	26	46.43		42	42.86		90	55.56		664	59.66		9	15.52	
**Reddit**																
	No	53	94.64	0.3220	92	93.88	0.0753	151	93.21	0.0044	1084	97.39	0.0927	55	94.83	0.3545
	Yes	3	5.36		6	6.12		11	6.79		29	2.61		3	5.17	
**Social media access**				0.3632			0.0044			< .0001			< .0001			< .0001
	Unlimited access	6	11.11		16	16.49		38	23.60		70	6.33		15	26.32	
	Limited access	17	31.48		41	42.71		54	33.54		374	33.85		13	22.81	
	No access	5	9.26		16	16.49		28	17.39		202	18.28		1	1.75	
	Unknown	26	48.15		24	24.74		41	25.47		459	41.54		28	49.12	
**Should be accessed at work for educational purposes only**																
	Agree	18	51.43	0.0181	55	77.46	0.1312	82	75.23	0.1700	535	69.75	0.7706	17	47.22	0.0031
	Disagree	17	48.57		16	22.54		27	24.77		232	30.25		19	52.78	
**Effective tool for educational purpose**																
	Agree	44	95.65	0.0678	82	97.62	0.0022	113	84.33	0.3958	752	85.84	0.1631	31	73.81	0.0125
	Disagree	2	4.35		2	2.38		21	15.67		124	14.16		11	26.19	
**Intended use of social media**																
**Social**																
	No	2	3.57	0.0041	11	11.22	0.0695	14	8.64	0.0010	112	10.06	< .0001	13	22.41	0.3809
	Yes	54	96.43		87	88.78		148	91.36		1001	89.94		45	77.59	
**Education**																
	No	32	57.14	0.5720	45	45.92	0.0019	78	48.15	0.0005	662	59.48	0.1216	32	55.17	0.3744
	Yes	24	42.86		53	54.08		84	51.85		451	40.52		26	44.83	
**Others**				0.8960												
	No	44	78.57		72.00	73.47	0.2805	107	66.05	0.0001	855	76.82	0.1417	45	77.59	0.9594
	Yes	12	21.43		26.00	26.53		55	33.95		258	23.18		13	22.41	
**Platform use for education**				** **	** **	** **	** **	** **	** **	** **	** **	** **	** **	** **	** **	** **
**Facebook**				0.1912												
	No	52	92.86		83	84.69	0.4628	141	87.04	0.9784	962	86.43	0.2395	46	79.31	0.0714
	Yes	4	7.14		15	15.31		21	12.96		151	13.57		12	20.69	
**Twitter**				< .0001												
	No	42	75.00		85	86.73	0.0527	108	66.67	< .0001	1072	96.32	< .0001	42	72.41	< .0001
	Yes	14	25.00		13	13.27		54	33.33		41	3.68		16	27.59	
**LinkedIn**																
	No	51	91.07	0.9461	73	74.49	< .0001	130	80.25	< .0001	1031	92.63	0.0002	53	91.38	0.8796
	Yes	5	8.93		25	25.51		32	19.75		82	7.37		5	8.62	
**Snapchat**																
	No	56	100.00	0.5138	98	100.00	0.3814	162	100.00	0.2503	1103	99.10	0.2451	58	100.00	0.5061
	Yes	0	0.00		0	0.00		0	0.00		10	0.90		0	0.00	
**Google+**																
	Yes	52	92.86	0.4664	90	91.84	0.1464	157	96.91	0.2296	1052	94.52	0.2467	56	96.55	0.5709
	No	4	7.14		8	8.16		5	3.09		61	61.00		2	3.45	
**Instagram**				0.1668												
	Yes	52	92.86		94	95.92	0.8411	156	96.30	0.9962	1067	95.87	0.1895	58	100.00	0.128
	No	4	7.14		4	4.08		6	3.70		46	4.13		0	0.00	
**Pinterest**				0.0122												
	Yes	53	94.64		83	84.69	0.4734	147	90.74	0.0023	856	76.91	< .0001	58	100.00	0.0003
	No	3	5.36		15	15.31		15	9.26		257	23.09		0	0.00	
**Reddit**				0.2246												
	Yes	54	96.43		95	96.94	0.2260	156	96.30	0.0226	1098	98.65	0.2713	58	100.00	0.3257
	No	2	3.57		3	3.06		6	3.70		15	1.35		0	0.00	

**Fig 2:**
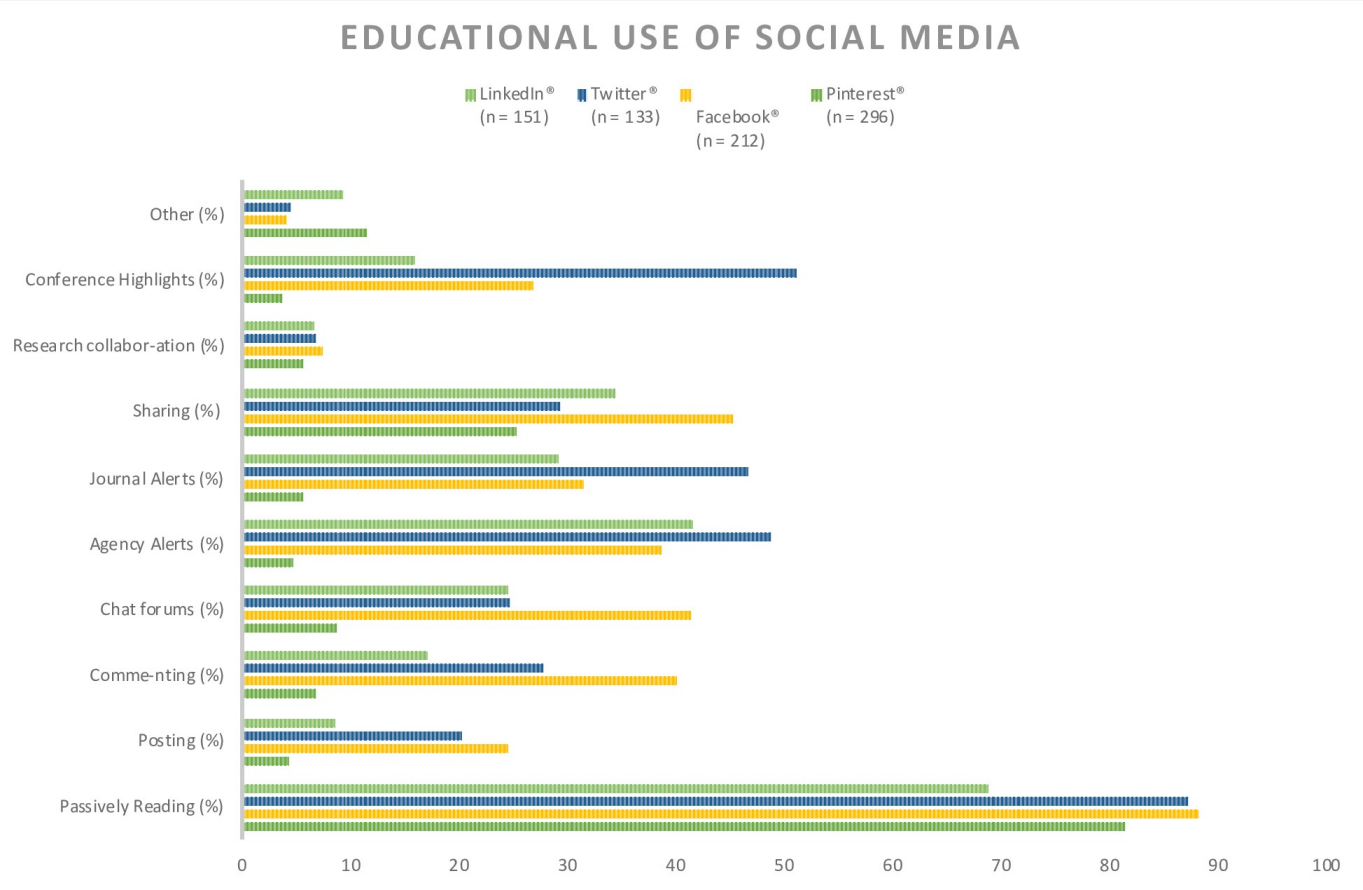
Percentage of respondents use of social media platforms* for particular educational purposes. *Percentage excluded of other platforms with n < 100.

Passively reading information was the primary way respondents used social media for educational purposes. Twitter® users reported following conference highlights (51%), health agency alerts (48%) and journal article alerts (46%) for educational purposes. About 25% of Facebook® users reported educational use in 8 of the areas evaluated. Less than 10% of respondents reported social media for research collaborations (Fig 2). Regarding social media access at work, unknown access was the most frequent answer (Table 1). Those with the most unlimited access amongst their profession were physicians (26%). Administrators reported having the most limited access (43%). The majority of administrators (77%), pharmacists (75%), nurses (70%), and residents (51%) all agreed that access to social media at work should be restricted for educational purposes only (Table 2). Additionally, the majority (59.7%) of respondents use their personal phone/computer to access social media at work (Table 1).

The propensity score weighted multivariable analyses compared a specific variable to all others regarding their likelihood of agreeing with two Likert style questions (i.e. answering strongly agree and agree). Residents, physicians, and those with unlimited access were more likely to disagree that social media access at work should be restricted for educational purposes only (OR 0.49 (95% CI 0.29 to 0.83), 0.50 (95% CI 0.31 to 0.81), 0.31 (95% CI 0.22 to 0.43) respectively). Those in the age group 40–49 also disagreed with this statement (OR 0.71 (95% CI 0.53 to 0.95) (Table 3). Respondents aged 20–29 were 43% more likely to agree that social media access at work should be restricted for educational purposes (OR 1.43 (95% CI 1.03 to 1.98)) (Table 4). Residents, who were primarily aged 20–29 (67%) responded that they use social media for social reasons more often compared to educational purposes (Table 3). Among physicians, 78% use social media for social reasons (Table 2). The age group of 40–49 was mainly comprised of nurses who disagree with allowing access to social media at work for educational purposes only. Administrators, pharmacists, and those with limited access were more likely to agree that social media use should be restricted to educational purposes at work (OR 2.09 (95% CI 1.31 to 3.34), OR 1.97 (95% CI 1.24 to 3.11), OR 1.30 (95% CI 1.02 to 1.66) respectively) (Table 4).

**Table 3:** Respondents’ attitudes regarding use of social media platforms.

Question	Strongly Agree	Agree	Neither Agree or Disagree	Disagree	Strongly Disagree
**Regardless of your current use of social media, access to social media should be accessible at work for educational purposes ONLY for you and/or your colleagues (n = 1,496)**	133 (8.9%)	585 (39.1%)	463 (30.9%)	246 (16.4%)	69 (4.6%)
**Access to social media at work would act as a useful marketing tool (n = 1,488)**	240 (16.1%)	672 (45.2%)	381 (25.6%)	142 (9.5%)	53 (3.6%)
**Access to social media at work would improve efficiency for you (n = 1,502)**	51 (3.4%)	234 (15.6%)	574 (28.2%)	500 (33.3%)	27 (1.8%)
**Access to social media at work would increase timeliness of healthcare information (n = 1,497)**	92 (6.1%)	354 (23.6%)	517 (34.5%)	414 (27.7%)	210 (8.0%)
**Access to social media at work would be/is a distraction in the workplace (1,499)**	351 (23.4%)	697 (46.5%)	296 (19.7%)	128 (8.5%)	27 (1.8%)

**Table 4:** Multivariate results of social media access at work for education purposes only for you and/or your colleagues.

		OR	95% (CI)		P-Value
**Professional affiliation**					
Residents					
	No (Ref)				
	Yes	0.49	0.29	0.83	0.0082
Administrators					
	No (Ref)				
	Yes	2.09	1.31	3.34	0.0021
Pharmacist					
	No (Ref)				
	Yes	1.97	1.24	3.11	0.0039
Nurse					
	No (Ref)				
	Yes	0.98	0.66	1.44	0.8992
Physicians					
	No (Ref)				
	Yes	0.50	0.31	0.81	0.0048
**Social media access**					
	No Access (ref)				
	Unlimited Access	0.31	0.22	0.43	< .0001
	Limited Access	1.30	1.02	1.66	0.0362
	Unknown	0.81	0.59	1.10	0.1798
**Age**					
	> 50 (Ref)				
	20–29	1.43	1.03	1.98	0.0329
	30–39	1.29	0.97	1.70	0.0795
	40–49	0.71	0.53	0.95	0.0194
**Geography**					
	Others (Ref)				
	Wisconsin Schools	0.71	0.45	1.11	0.1286
	Georgia Schools	1.13	0.28	4.61	0.8669
	South Carolina Schools	0.63	0.40	0.99	0.0433
	Maryland Schools	1.02	0.25	4.25	0.9742

The second question analyzed by the multivariable analysis was regarding whether social media can be used as an effective tool for educational purposes. Pharmacists (OR 0.21, 95% CI 0.11 to 0.38), nurses (OR 0.31, 95% CI 0.17 to 0.56), and physicians (OR 0.17, 95% CI 0.09 to 0.33) all were in disagreement with this statement (Table 5). Among the pharmacist, nurse, and physician respondents that use social media, 52%, 41%, and 45% of them respectively, use it for educational purposes (Table 3). Administrators (OR 3.41, 95% CI 1.31 to 8.84), those with unlimited and limited access (OR 4.18, 95% CI 2.30 to 7.60 and OR 1.66, 95% CI 1.22 to 2.25 respectively), and those in the two age groups of 20–29 and 30–39 (OR 4.40, 95% CI 2.80 to 6.92 and OR 2.14 95% CI 1.53 to 3.01) all agreed with this statement (Table 5).

**Table 5:** Multivariate results of social media is an effective tool for educational purposes.

		OR	95% (CI)		P-Value
**Professional affiliation**					
Residents					
	No (Ref)				
	Yes	0.57	0.26	1.27	0.1676
Administrators					
	No (Ref)				
	Yes	3.41	1.31	8.84	0.0117
Pharmacist					
	No (Ref)				
	Yes	0.21	0.11	0.38	< .0001
Nurse					
	No (Ref)				
	Yes	0.31	0.17	0.56	0.0001
Physicians					
	No (Ref)				
	Yes	0.17	0.09	0.33	< .0001
**Social media access**					
	No Access (ref)				
	Unlimited Access	4.18	2.30	7.60	< .0001
	Limited Access	1.66	1.22	2.25	0.0012
	Unknown	0.71	0.49	1.02	0.0618
**Age**					
	> 50 (Ref)				
	20–29	4.40	2.80	6.92	< .0001
	30–39	2.14	1.53	3.01	< .0001
	40–49	1.22	0.87	1.71	0.2533
**Geography**					
	Others (Ref)				
	Wisconsin Schools	0.50	0.29	0.86	0.012
	Georgia Schools	0.18	0.05	0.71	0.0142
	South Carolina Schools	0.62	0.36	1.07	0.0871
	Maryland Schools	1.12	0.25	1.31	0.9766

## Discussion

Healthcare information is continuously updating, and the volume of newly added data has never been greater. Healthcare workers must be both creative and efficient in their methods for maintaining an updated database that is relevant to clinical practice. Our investigation aimed to express the views of healthcare professionals regarding the use of social media as a platform for healthcare education. A large percentage (43%) of the respondents reported using social media for educational purposes.

### Educational uses of social media

Sharing/exchanging ideas with other professionals, chat discussions, following conference highlights, and healthcare agency alerts were some of the ways respondents in this present study expressed their use of social media for educational purposes. This is similar to the findings of authors from the University of Scranton who highlighted 5 ways social media is used by healthcare professionals: sharing information, comparing and improving quality, training medical personnel, live updates during procedures, and communication through times of crisis [27]. Ventola and colleagues also described social media in healthcare use to include professional networking, professional education, organizational promotion, patient care, patient education, and public health programs [8]. There are many resources in the literature on this topic [28, 29]. Not surprisingly, those under 40 years of age strongly agreed that social media was an effective tool for educational purposes. Although, it is unclear why pharmacists, attending physicians and nurses in our present study disagreed that social media was an effective educational tool. Interestingly, Twitter® (61.9%) and Pinterest® (59.5%) had more low frequency users but were in the top group for social media accounts used for educational purposes. The effectiveness of social media has not been fully evaluated in the literature and may not correlate directly with social media use depending on certain platforms.

An increase in journal awareness is another way to utilize social media. O’Kelly and colleagues found that the presence of a Twitter® feed contributed to an increased impact factor (P = 0.017) in urological and pediatric journals from 2012–2016 [30]. Additionally, a new non-traditional metric of professional impact called Altmetric (https://www.altmetric.com) analyzes the penetration of a published article through social media and non-publisher or journal affiliated outlets. It provides an alternative way to view and measure the article’s activity outside of the journal’s impact factor [31–34]. Professional conferences increasingly utilize social media and designated hashtags to link and disseminate conference and other pertinent healthcare information. These topical hashtags have prompted numerous interactions on social media [35]. Our survey results were similar to several previous studies, demonstrating the multifunctional use of social media in healthcare for education. Although, in our study, research collaboration was not a common reason for social media use, interactions and relationships built over time may allow for future collaboration through more traditional means (e.g. meeting at conferences, email contact).

### Social media access at work

In general, our data show access to social media at work affected ideas regarding using social media for educational purposes. Those with unlimited access to social media were less likely to agree with the benefit of social media for education (OR = 0.30, P < 0.0001). We hypothesize this is due to the wording of the survey item. Since the item stated using social media for educational purposes only in the workplace, then those with unlimited access could have viewed this as a restriction to their current access. In contrast, those with limited access could have agreed with the statement because of more desired access at work. In today’s age of smart phones and data plans, it is safe to say this also could have affected these results.

### Nursing response impact

This study was impacted by a large percentage of nurse respondents (75%). Prior studies have shown an interest in electronic resources for educational purposes by nursing beginning as early as 1990 [36]. With the advancement of technology, nursing use of social media for healthcare education has become present with protocols, activities, and resources [37–39]. Rutledge et al designed a tool on social media for Doctor of Nursing Practice program students in rural health care [40]. Another survey-based study among first year nursing students demonstrated that 81% of students felt Twitter® was beneficial in increasing awareness of nursing issues [41]. With nurses being the largest group healthcare members who responded to this survey, this could also explain why Pinterest® was in the top group of social media platforms used for healthcare education since nursing responded to using Pinterest® the most (60%) out of the practitioners. One study analyzing the accuracy of information on Pinterest® for psychogenic non-epileptic seizures (PNES) found 87.7% of the 57 pins analyzed reporting at least one factor indicative of PNES [42] Pinterest® has also aided nursing faculty in preparation and educational activities [43]. Targeting social media platforms like Twitter® and Pinterest® in future studies may be beneficial to analyze potential correlation between frequency of social media use and use for educational purposes. Our results having a high response-rate by nurses could be attributed to the amount of nursing education and connectivity with social media in this field. Results were adjusted by a propensity score in order to reduce bias in the data.

### Healthcare administrator perspective

Healthcare administrators (n = 98, 5.96%) in this study were more than 4 times as likely to agree that social media could be an effective educational tool (Table 5). They were also 59% more likely to agree with allowing social media use for educational purposes in the workplace (Table 2). This was interesting since a higher percentage of respondents answered that they had limited or no access at work. The Sentinel Watch, a blog by American Sentinel University that offers degrees in nursing and healthcare management, encourages hospital administrators to have a presence on social media [44]. In addition, some healthcare organizations, including the American College of Clinical Pharmacy and American Association of Colleges of Pharmacy, have published reports or conducted webinars to provide advice on the importance of and how to build a digital brand through social media [45–47]. Social media is a platform that could be utilized to market services of the healthcare system or to provide access for complaints or appraisals in a quick manner [48]. There are many further options to utilize social media as a resource to reach patients and deliver patient care, and while not educational per se, it does encourage social media activity among healthcare administrators [49–51]. Because of this open interaction, it is imperative to note the potential dangers of social media which include poor quality of information, damage to professional image, breaches of patient privacy (e.g. HIPAA), violation of the patient-healthcare practitioner boundary, licensing issues, and legal issues. The necessity of professional guidelines for the use of social media by institutions needs to be stressed [8]. Interestingly, a large percentage of respondents (40%) did not know their workplace social media policy (Table 1). However, this mirrors previously published data where a similar percentage of healthcare workers were unaware of their workplace policy [52]. These are also important to recognize if trying to implement social media for educational purposes into clinical practice, as there may be significant institutional or operational barriers. Only 31% of healthcare organizations have specific social media guidelines for writing or posting social media content. However, 26% of U.S. hospitals are already utilizing social media in some form for education [53]. On the other hand, 70% of respondents in this study, stated that access to social media at work would be or currently is a distraction. As mentioned above, distraction is another consideration that is noted as a risk in previous literature explaining how to use social media in the workplace [8].

### Strengths and limitations

The strengths of this study include the different sites represented which allowed for both a large sample size and responses from different regions in the US and professions. All the survey questions collected views on multiple social media platforms for educational use. The limitations of this study include the issues regarding none of the survey questions were required to answer in order to submit the survey. This limited some responses such as the primary profession indication and social media account use. The primary responses of “administrative roles” and “other” could have been confusing for the respondent and should have been defined. This confusion could have included unintended and/or excluded eligible participants. Throughout the distribution of the survey, not all sites received the survey on the exact same day, leading to the survey being available from January 22 through May 1, 2018. Additionally, an exact record of how many individuals received the survey emails was not able to be recorded. Email listservs were utilized which made it difficult to track the total number of potential participants. Responder bias also played a factor in this study. Those who are more engaged in social media may have been more likely to complete the study. This was accounted for by the propensity score weighted multivariable analyses. As mentioned previously, the large percentage of nurse respondents may have biased the results towards nursing profession, however, this was accounted for with propensity scoring. Based on these results and others, one future area of study will be to further evaluate the effectiveness of social media as an educational tool among healthcare practitioners.

## Conclusions

The majority of healthcare workers in this study believe social media can be an effective tool for healthcare education. Understanding how to best leverage social media in this capacity may vary for each profession, since many healthcare practitioners currently use social media in various ways. Future studies should analyze how to utilize these platforms efficiently and effectively for healthcare education. Additional studies are also needed to better understand social media education platforms for physicians and healthcare administrators. These data can serve as a source for individuals who may want to propose social media as an avenue to obtain or provide healthcare-related education.

## Supporting information

S1 File Views of social media for educational use in healthcare survey. (PDF)

S1 Table Respondents’ attitudes regarding use of social media platforms. (DOCX)

S2 Table Frequency of social media use. (DOCX)

S3 Table Additional survey question responses. (DOCX)
