# Supplementary material for: Healthcare practitioners’ views of social media as an educational resource
Source: PLoS One. 2020 Feb 6;15(2):e0228372. doi: 10.1371/journal.pone.0228372 (PMC7004337; doi:10.1371/journal.pone.0228372)
Supplement: S1 File — (PDF) [file pone.0228372.s001.pdf]

# Views of Social Media for Educational Use in Healthcare

The goal of this survey is to gain insight regarding the perception of social media and its role in health care. This is a voluntary survey aimed at the healthcare professional practioner/academia/administration population. You are being asked to partcipate in this study because you meet these requirements. Please complete the survey below to all questions that apply to you and your social media use. Thank you for your time.

Be sure to enter your email at the end of the survey for a chance to win a $50 Amazon gift card.

Definitions:

Social use of social media: any action related to personal interests/information outside of the healthcare field (e.g. posting pictures from a dinner party, liking posts of movies coming soon).

Educational use of social media: defined as anything regarding the healthcare field that you deem as knowledgable and useful information for the field (e.g. accessing journal articles, reading drug updates).

What is/are your primary professional role(s)? (Please select all that apply)

- ☐ Student
- ☐ Resident
- ☐ Administrator
- ☐ Pharmacist
- ☐ Registered Nurse
- ☐ Attending Physician
- ☐ Physician Assistant
- ☐ Nurse Practitioner
- ☐ Other

What type of field would best describe your administrative position?

- ◯ Academia (e.g. department chair)
- ◯ Chief officer (e.g. CEO, CFO, COO)
- ◯ Manager (e.g. department/site manager, coordinator)
- ◯ Other

If other, please explain ______________________________

Which social media account(s) do you have? (Please select all that apply)

- ☐ Facebook&#174
- ☐ Twitter&#174
- ☐ Linkedin&#174
- ☐ Snapchat&#174
- ☐ Google+&#174
- ☐ Instagram&#174
- ☐ Pinterest&#174
- ☐ Reddit&#174
- ☐ Other
- ☐ None

If other please explain. ______________________________

What is/are your main reason(s) you do not have a social media account? (Please select all that apply)

- ☐ Do not have time for it
- ☐ Privacy concern
- ☐ Lack of interest
- ☐ It is not user friendly
- ☐ Other

If other please explain. ______________________________

**How often do you use the following social media platform(s)?**

| | Have account but never on | monthly | weekly | multiple times per week | daily | multiple times per day |
|---|---|---|---|---|---|---|
| Facebook&#174 | ○ | ○ | ○ | ○ | ○ | ○ |
| Twitter&#174 | ○ | ○ | ○ | ○ | ○ | ○ |
| Linkedin&#174 | ○ | ○ | ○ | ○ | ○ | ○ |
| Snapchat&#174 | ○ | ○ | ○ | ○ | ○ | ○ |
| Google+&#174 | ○ | ○ | ○ | ○ | ○ | ○ |
| Instagram&#174 | ○ | ○ | ○ | ○ | ○ | ○ |
| Pinterest&#174 | ○ | ○ | ○ | ○ | ○ | ○ |
| Reddit&#174 | ○ | ○ | ○ | ○ | ○ | ○ |
| Other | ○ | ○ | ○ | ○ | ○ | ○ |

**How would you best describe your intended use of social media platforms?**

| | Social | Educational | Other |
|---|---|---|---|
| Facebook&#174 | ☐ | ☐ | ☐ |
| Twitter&#174 | ☐ | ☐ | ☐ |
| Linkedin&#174 | ☐ | ☐ | ☐ |
| Snapchat&#174 | ☐ | ☐ | ☐ |
| Google+&#174 | ☐ | ☐ | ☐ |
| Instagram&#174 | ☐ | ☐ | ☐ |
| Pinterest&#174 | ☐ | ☐ | ☐ |
| Reddit&#174 | ☐ | ☐ | ☐ |
| Other | ☐ | ☐ | ☐ |

If other please explain. ______________________________

How are you using Facebook&#174 for educational reasons? (Please select all that apply)

- ☐ Passively reading information
- ☐ Posting original content
- ☐ Commenting on existing content
- ☐ Educational chat/discussions forums
- ☐ Healthcare agency alerts (e.g. FDA, CDC)
- ☐ Healthcare Journal alerts (e.g. JAMA, table of contents)
- ☐ Sharing/Exchanging ideas with other professionals
- ☐ Research collaboration
- ☐ Following conference highlights (e.g. event hashtags)
- ☐ Other

If other please explain. ______________________________

How are you using Twitter&#174 for educational reasons? (Please select all that apply)

- ☐ Passively reading information
- ☐ Posting original content
- ☐ Commenting on existing content
- ☐ Educational chat/discussions forums
- ☐ Healthcare agency alerts (e.g. FDA, CDC)
- ☐ Healthcare Journal alerts (e.g. JAMA, table of contents)
- ☐ Sharing/Exchanging ideas with other professionals
- ☐ Research collaboration
- ☐ Following conference highlights (e.g. event hashtags)
- ☐ Other

If other please explain. __________________________________

How are you using Linkedin&#174 for educational reasons? (Please select all that apply)

- ☐ Passively reading information
- ☐ Posting original content
- ☐ Commenting on existing content
- ☐ Educational chat/discussions forums
- ☐ Healthcare agency alerts (e.g. FDA, CDC)
- ☐ Healthcare Journal alerts (e.g. JAMA, table of contents)
- ☐ Sharing/Exchanging ideas with other professionals
- ☐ Research collaboration
- ☐ Following conference highlights (e.g. event hashtags)
- ☐ Other

If other please explain. __________________________________

How are you using Snapchat&#174 for educational reasons? (Please select all that apply)

- ☐ Passively reading information
- ☐ Posting original content
- ☐ Commenting on existing content
- ☐ Educational chat/discussions forums
- ☐ Healthcare agency alerts (e.g. FDA, CDC)
- ☐ Healthcare Journal alerts (e.g. JAMA, table of contents)
- ☐ Sharing/Exchanging ideas with other professionals
- ☐ Research collaboration
- ☐ Following conference highlights (e.g. event hashtags)
- ☐ Other

If other please explain. __________________________________

How are you using Google+&#174 for educational reasons? (Please select all that apply)

- ☐ Passively reading information
- ☐ Posting original content
- ☐ Commenting on existing content
- ☐ Educational chat/discussions forums
- ☐ Healthcare agency alerts (e.g. FDA, CDC)
- ☐ Healthcare Journal alerts (e.g. JAMA, table of contents)
- ☐ Sharing/Exchanging ideas with other professionals
- ☐ Research collaboration
- ☐ Following conference highlights (e.g. event hashtags)
- ☐ Other

If other please explain. __________________________________

How are you using Instagram&#174 for educational reasons? (Please select all that apply)

- ☐ Passively reading information
- ☐ Posting original content
- ☐ Commenting on existing content
- ☐ Educational chat/discussions forums
- ☐ Healthcare agency alerts (e.g. FDA, CDC)
- ☐ Healthcare Journal alerts (e.g. JAMA, table of contents)
- ☐ Sharing/Exchanging ideas with other professionals
- ☐ Research collaboration
- ☐ Following conference highlights (e.g. event hashtags)
- ☐ Other

If other please explain. __________________________________

How are you using Pinterest&#174 for educational reasons? (Please select all that apply)

- ☐ Passively reading information
- ☐ Posting original content
- ☐ Commenting on existing content
- ☐ Educational chat/discussions forums
- ☐ Healthcare agency alerts (e.g. FDA, CDC)
- ☐ Healthcare Journal alerts (e.g. JAMA, table of contents)
- ☐ Sharing/Exchanging ideas with other professionals
- ☐ Research collaboration
- ☐ Following conference highlights (e.g. event hashtags)
- ☐ Other

If other please explain. __________________________________

How are you using Reddit&#174 for educational reasons? (Please select all that apply)

- ☐ Passively reading information
- ☐ Posting original content
- ☐ Commenting on existing content
- ☐ Educational chat/discussions forums
- ☐ Healthcare agency alerts (e.g. FDA, CDC)
- ☐ Healthcare Journal alerts (e.g. JAMA, table of contents)
- ☐ Sharing/Exchanging ideas with other professionals
- ☐ Research collaboration
- ☐ Following conference highlights (e.g. event hashtags)
- ☐ Other

If other please explain. __________________________________

How are you using other for educational reasons? (Please select all that apply)

- ☐ Passively reading information
- ☐ Posting original content
- ☐ Commenting on existing content
- ☐ Educational chat/discussions forums
- ☐ Healthcare agency alerts (e.g. FDA, CDC)
- ☐ Healthcare Journal alerts (e.g. JAMA, table of contents)
- ☐ Sharing/Exchanging ideas with other professionals
- ☐ Research collaboration
- ☐ Following conference highlights (e.g. event hashtags)
- ☐ Other

If other please explain. __________________________________

A social media presence makes an employee valuable in the workplace.

- ◯ Strongly Agree
- ◯ Agree
- ◯ Neither agree or disagree
- ◯ Disagree
- ◯ Strongly disagree

Engagement in social media makes me a better healthcare practitioner/provider.

- ◯ Strongly Agree
- ◯ Agree
- ◯ Neither agree or disagree
- ◯ Disagree
- ◯ Strongly disagree

Social media can be used as an effective tool for educational purposes.

- ◯ Strongly Agree
- ◯ Agree
- ◯ Neither agree or disagree
- ◯ Disagree
- ◯ Strongly disagree

Please select why you do not view social media as an effective tool for educational purposes. (Please select all that apply)

- ☐ Don't see value
- ☐ Question validity of source
- ☐ Restricted access at place of employment
- ☐ Legal concerns
- ☐ No compensation for giving advice/recommendations on social media
- ☐ Other

If other please explain. __________________________________

What best describes how you access social media at work?

- ◯ Personal phone/computer
- ◯ Work phone/computer
- ◯ I do not access social media at my place of employment
- ◯ Other

If other please explain. __________________________________

What type of social media access is available at your workplace?

- ◯ Unlimited Access
- ◯ Limited Access (some restrictions)
- ◯ No Access
- ◯ I dont know

If restrictions apply to social media access in your workspace, please explain. __________________________________

Is there a current policy that blocks social media access at your place of employment?

- ◯ Yes
- ◯ No

Regardless of your current use of social media, access to social media should be accessible at work for you and/or your colleagues.

- ◯ Strongly Agree
- ◯ Agree
- ◯ Neither agree or disagree
- ◯ Disagree
- ◯ Strongly disagree

Regardless of your current use of social media, access to social media should be accessible at work for educational purposes ONLY for you and/or your colleagues.

- ◯ Strongly Agree
- ◯ Agree
- ◯ Neither agree or disagree
- ◯ Disagree
- ◯ Strongly disagree

**Access to social media platforms at your workplace would/does:**

| | Strongly agree | Agree | Neither agree or disagree | Disagree | Strongly disagree |
|---|---|---|---|---|---|
| Improve efficiency for you | ◯ | ◯ | ◯ | ◯ | ◯ |
| Improve efficiency for colleagues | ◯ | ◯ | ◯ | ◯ | ◯ |
| Increase timeliness of healthcare information | ◯ | ◯ | ◯ | ◯ | ◯ |
| Act as a useful marketing tool | ◯ | ◯ | ◯ | ◯ | ◯ |

Social media access would be/is a distraction in the workplace.
- ◯ Strongly Agree
- ◯ Agree
- ◯ Neither agree or disagree
- ◯ Disagree
- ◯ Strongly disagree

Age (years)
- ◯ 20-29
- ◯ 30-39
- ◯ 40-49
- ◯ 50-59
- ◯ 60-69
- ◯ 70-79
- ◯ > 80

Please select your primary place of employment.
- ◯ University of Wisconsin Health
- ◯ University of Wisconsin- Madison School of Medicine
- ◯ University of Wisconsin- Madison School of Pharmacy
- ◯ University of Wisconsin- Madison Physician Assistant Program
- ◯ University of Wisconsin- Madison School of Nursing
- ◯ University of Georgia College of Pharmacy
- ◯ Palmetto Health Hospital System (SC)
- ◯ University of South Carolina College of Pharmacy
- ◯ University of South Carolina School of Medicine
- ◯ University of South Carolina College of Nursing
- ◯ University of South Carolina Physician Assistant Program
- ◯ University of Maryland Medical Center
- ◯ University of Maryland School of Pharmacy
- ◯ University of Maryland School of Medicine
- ◯ University of Maryland School of Nursing
- ◯ Other

If other please explain. ________________________________

How many years has it been post terminal degree?
- ◯ 5 or less
- ◯ 6-10
- ◯ 11-15
- ◯ 16-20
- ◯ 21+

Do you have any other input you would like to share about using Social Media as a means for educational information use? ________________________________________

Are you interested in further resources on social media as an educational tool?
- ◯ Yes
- ◯ No

Link to other relative studies:

[Attachment: "Social Media in Healthcare .pdf"]

In order to be included in the drawing for a chance to win 1 of 4 $50 Amazon gift cards, please enter your email here. Your email will not be included with the analysis of the study results, nor will it affect your chances of winning a gift card. __________________________________
