# Supplementary material for: Healthcare practitioners’ views of social media as an educational resource
Source: PLoS One. 2020 Feb 6;15(2):e0228372. doi: 10.1371/journal.pone.0228372 (PMC7004337; doi:10.1371/journal.pone.0228372)
Supplement: S1 Table — (DOCX) [file pone.0228372.s002.docx]

**S1 Table: Respondents’ attitudes regarding use of social media platforms**

| **Question*** | **Strongly**  **Agree** | **Agree** | **Neither Agree or Disagree** | **Disagree** | **Strongly Disagree** |
| --- | --- | --- | --- | --- | --- |
| **Social media can be used as an effective tool for educational purposes (n = 1,504)** | 228  (15.2%) | 813  (54.1%) | 303  (20.1%) | 131  (8.7%) | 29  (1.9%) |
| **Regardless of your current use of social media, access to social media should be accessible at work for educational purposes ONLY for you and/or your colleagues**  **(n = 1,496)** | 133 (8.9%) | 585  (39.1%) | 463  (30.9%) | 246  (16.4%) | 69  (4.6%) |
| **Access to social media at work would act as a useful marketing tool**  **(n = 1,488)** | 240 (16.1%) | 672  (45.2%) | 381  (25.6%) | 142  (9.5%) | 53  (3.6%) |
| **Access to social media at work would improve efficiency for you**  **(n = 1,502)** | 51  (3.4%) | 234  (15.6%) | 574  (28.2%) | 500  (33.3%) | 27  (1.8%) |
| **Access to social media at work would improve efficiency for your colleagues (n = 1,500)** | 49  (3.3%) | 211  (14.1%) | 579  (38.6%) | 491  (32.7%) | 170  (11.3%) |
| **Access to social media at work would increase timeliness of healthcare information**  **(n = 1,497)** | 92  (6.1%) | 354  (23.6%) | 517  (34.5%) | 414  (27.7%) | 210  (8.0%) |
| **Access to social media at work would be/is a distraction in the workplace (n = 1,499)** | 351 (23.4%) | 697  (46.5%) | 296  (19.7%) | 128  (8.5%) | 27  (1.8%) |
| **Engagement in social media makes me a better healthcare practitioner/provider**  **(n = 1,372)** | 72  (5.2%) | 243  (17.7%) | 726  (52.9%) | 245  (17.9%) | 86  (6.3%) |

*Excluded responses that were n < 100
