# Supplementary material for: Healthcare practitioners’ views of social media as an educational resource
Source: PLoS One. 2020 Feb 6;15(2):e0228372. doi: 10.1371/journal.pone.0228372 (PMC7004337; doi:10.1371/journal.pone.0228372)
Supplement: S2 Table — (DOCX) [file pone.0228372.s003.docx]

**S2 Table: Frequency of social media use**

| **Social media platform*** | **Have account but never on** | **Monthly** | **Weekly** | **Multiple times per week** | **Daily** | **Multiples times per day** |
| --- | --- | --- | --- | --- | --- | --- |
| **Facebook®**  **(n = 1,298)** | **42 (3.2%)** | **62 (4.8%)** | **99 (7.6%)** | **138 (10.6%)** | **407 (31.4%)** | **550 (42.4%)** |
| **Pinterest®**  **(n = 825)** | **67 (8.1%)** | **221 (26.8%)** | **203 (24.6%)** | **210 (25.5%)** | **79 (9.6%)** | **45 (5.5%)** |
| **Instagram®**  **(n = 789)** | **52 (6.6%)** | **92 (11.7%)** | **92 (11.7%)** | **98 (12.4%)** | **207 (26.2%)** | **248 (31.4%)** |
| **Snapchat®**  **(n = 632)** | **53 (8.4%)** | **56 (8.9%)** | **67 (10.6%)** | **88 (13.9%)** | **159 (25.2%)** | **209 (33.1%)** |
| **LinkedIn®**  **(n = 461)** | **178 (38.6%)** | **167 (36.2%)** | **67 (14.5%)** | **28 (6.1%)** | **13 (2.8%)** | **8 (1.7%)** |
| **Twitter®**  **(n = 415)** | **125 (30.1%)** | **69 (16.6%)** | **63 (15.2%)** | **55 (13.3%)** | **52 (12.5%)** | **51 (12.3%)** |
| **Snapchat®**  **(n = 632)** | **53 (8.4%)** | **56 (8.9%)** | **67 (10.6%)** | **88 (13.9%)** | **159 (25.2%)** | **209 (33.1%)** |
| **Google+®**  **(n = 280)** | **88 (31.4%)** | **47 (16.8%)** | **31 (11.1%)** | **35 (12.5%)** | **46 (16.4%)** | **33 (11.8%)** |

***Excluded platforms with n < 100**
