# Supplementary material for: Healthcare practitioners’ views of social media as an educational resource
Source: PLoS One. 2020 Feb 6;15(2):e0228372. doi: 10.1371/journal.pone.0228372 (PMC7004337; doi:10.1371/journal.pone.0228372)
Supplement: S3 Table — (DOCX) [file pone.0228372.s004.docx]

**S3 Table: Additional survey question responses**

| **Survey question** | **Survey answers** | **Response n, (%)** |
| --- | --- | --- |
| **What type of filed would best describe your administrative position (n = 96)** | **Manager (e.g. department/site manager, coordinator)**  **Other**  **Academia (e.g. department chair)**  **Chief officer (e.g. CEO, CFO, COO)** | **55 (57.3%)**  **34 (35.4%)**  **4 (4.2%)**  **3 (3.1%)** |
| **What is/are your main reason(s) you do not have a social media account? (Please select all that apply) (n = 90)** | **Privacy concern**  **Lack of interest**  **Do not have time for it**  **Other**  **It is not user friendly** | **53 (58.9%)**  **51 (57.8%)**  **48 (53.3%)**  **6 (6.7%)**  **1 (1.1%)** |
| **Please select why you do not view social media as an effective tool for educational purposes (Please select all that apply)**  **(n = 457)** | **Question validity of source**  **Legal concerns**  **Restricted access at place of employment**  **Don’t see value**  **No compensation for giving advice/recommendations**  **Other** | **360 (78.8%)**  **163 (35.7%)**  **115 (25.2%)**  **105 (23.0%)**  **44 (9.6%)**  **38 (8.3%)** |
| **What best describes how you access social media at work (n = 1,408)** | **Personal phone/computer**  **I do not access social media at my place of employment**  **Work phone/computer**  **Other** | **840 (59.7%)**  **506 (35.9%)**  **41 (2.9%)**  **21 (1.5%)** |
